# Birth Weight, Growth and Feeding Pattern in Early Infancy Predict Overweight/Obesity Status at Two Years of Age: A Birth Cohort Study of Chinese Infants

**DOI:** 10.1371/journal.pone.0064542

**Published:** 2013-06-05

**Authors:** Jianduan Zhang, John H. Himes, Yuan Guo, Jingxiong Jiang, Liu Yang, Qiaozhen Lu, Haiyan Ruan, Shuhua Shi

**Affiliations:** 1 Department of Woman and Child’s Care and Adolescence Health, School of Public Health, Tongji Medical College, Huazhong University of Science and Technology, Wuhan, Hubei, China; 2 Department of Epidemiology and Community Health, School of Public Health, University of Minnesota, Minneapolis, Minnesota, United States of America; 3 Department of Child Health, National Center of Women and Children’s Health Care, Chinese Center for Disease Control and Prevention, Beijing, China; 4 Department of Maternal Health, Shenyang Municipal Maternal and Child Health Care Center, Shenyang, Liaoning, China; 5 Department of Child Health, Haizhu Maternal and Child Health Hospital, Guangzhou, Guangdong, China; 6 Department of Child Health, Panyu District Maternal and Child Health Hospital, Guangzhou, Guangdong, China; University of Missouri-Kansas City, United States of America

## Abstract

**Objectives:**

To investigate the early determinants of overweight and obesity status at age two years.

**Methods:**

A total of 1098 healthy neonates (563 boys and 535 girls) were involved in this community-based prospective study in China. Data on body weight and length were collected at birth, the 3^rd^ and 24^th^ month. A self-administered questionnaire was used to collect data on social demography and feeding patterns of children, etc. Three multivariable logistic regression models were employed to make various comparisons of weight status, i.e., model 1 (obesity vs. non-obesity), model 2 (combined overweight and obesity vs. normal weight, and model 3 (obesity, overweight and normal weight).

**Results:**

Prevalences of overweight/obesity (95^th^ >BMI ≥85^th^ p and BMI ≥95^th^ p, referring to WHO BMI standards) at 2 years of age are 15.8%/11.2% for boys and 12.9%/9.0% for girls, respectively. Being born with macrosomia (OR: 1.80–1.88), relatively greater BMI increment in the first 3 months (OR: 1.15–1.16) and bottle emptying by encouragement at age two (OR: 1.30–1.57) were found in all three models to be significant risk factors for higher BMI status at 2 years. Pre-pregnancy maternal BMI (OR: 1.09–1.12), paternal BMI (OR: 1.06), and mixed breastfeeding (OR: 1.54–1.57) or formula feeding (OR: 1.90–1.93) in the first month were identified as significant in models 2 and 3. Child-initiated bottle emptying at age two was observed to increase the risk of obesity by 1.31 times but only in model 1.

**Conclusion:**

Fetal and early postnatal growth and feeding pattern appear to have significant impacts on early childhood overweight and obesity status independent of parental BMI. Policy-based and multidisciplinary approaches to promote breastfeeding and enhancement of feeding skills of care takers may be promising intervention strategies.

## Introduction

It is well documented that the prevalences of childhood overweight and obesity have been accelerating in different settings and have become a critical world-wide public health concern [1]–[4]. The prevalence of overweight and obesity among preschool children increased 48% and 65%, respectively, in developed and developing countries between 1990 and 2010 [5].

The economic boom in China has been accompanied with some unintended ‘by-products’, e.g., in urban China the prevalence of obesity in preschool children increased from 1.5% in 1989 to 12.6% in 1997, and that of overweight increased from 14.6% to 28.9% in the same period [6], [7]. The combined overweight and obesity prevalence, and obesity-only prevalence among school age children reached 34.2% and 30.3%, respectively in 2005 [8]. These prevalences are now comparable with or even exceed those in some western countries [9], [10].

Obese children are at elevated risks of many health conditions, e.g., type 2 diabetes mellitus, high blood pressure, high cholesterol and other cardiovascular diseases [11]–[14], along with greater health-care costs in higher BMI groups [15]. They are more likely to suffer from adverse emotional and social conditions [16]. Furthermore, the accumulating excessive adiposity is very likely to track into the adulthood and is associated with greater middle-age mortality and morbidity [17]–[19], and consequently leads to enormous health-related economic burden.

Infancy is understood as a critical period for the development of obesity many reasons, but primarily because infants are experiencing food transitions, establishment of eating habits, and too often, the early development of excess adiposity. Recently, more evidence has become available regarding the associations of early weight status and rapid growth, with obesity and related problems in later life [20] [21]. For instance, Harrington’s study showed that more than half of the overweight children aged 2 to 20 years became overweight before the age of two [21].

So far, there have been no universally effective intervention strategies or treatments for reducing the prevalence of obesity in populations. Prevention of childhood overweight at an early age, i.e., as early as two years, therefore becomes of utmost importance [22]. Consequently, to develop possible early intervention strategies, the evaluation of associations between early modifiable determinants and overweight and obesity status in infancy is an urgent need.

Our previous studies have demonstrated the protective effects of breastfeeding for both high weight gain and high BMI status at the age of three months in a large sample of Chinese children [23]. In this paper, we present data from our evaluation of new data on same cohort concerning the longer-term impacts of early feeding patterns on BMI status at two years of age. Also presented in this paper are the impacts of other potentially modifiable factors such as BMI increment in the first three months.

## Materials and Methods

A total of 2, 217 newborns were recruited at the initiation of the cohort in three cities (1,154 boys and 1,063 girls) through a multistage sampling scheme. The three cities were selected to represent three distinct administrative and geographic regions of China: Shenyang, Wuhan and Guangzhou, the capital cities of Liaoning (Northern China), Hubei (Central China) and Guangdong (Southern China) provinces. These are relatively affluent cities and the prevalences of obesity are thought to be relatively high. The participants were recruited within the first month following birth, and the children were followed up until two years of age. The cohort yielded a total of 1,098 children with complete data at birth and months 1, 3, and 24, who were included in these analyses. The number of children with available measurements at the corresponding age points was 2,076, 1,801 and 1,179. The reasons for the reduction in numbers over time included the parents being too busy for follow-up visits to the participating Community Health Service Centers/Stations (CHSC), children’s sickness, bad weather, travelling out of the town, or drop-out from the study. No significant differences were identified between originally recruited children and those who had complete data measurements in their parental social-demographic and anthropometric variables, and in their birth situation, except for the age of father (31.3 vs. 31.0 years). Therefore, possible bias due to drop-outs is believed to be limited.

Within each of the three urban areas, CHSC were randomly selected. The participant infants were then randomly selected from among newborns to local residents that were registered and who would receive health surveillance and routine follow-up. All protocol-required procedures were standardized across the three field sites and data collectors were trained and monitored centrally. Informed consent was obtained from the parents.

Birth weight (BW) and crown-heel length at birth were obtained from the Perinatal Health Booklet (PHB), which was kept by individual parents, and included health information recorded related to pregnancy from the first prenatal checkup until 42 days following delivery. These data were abstracted from booklets by uniformly trained health staff. Birth weight (BW) is initially presented here as three categories, i.e. low birth weight (BW, 2500 g), normal weight, and macrosomia. For the final regression analysis, however, BW was dichotomized into non-macrosomia (BW<4000 g) and macrosomia BW≥4000 g) because of the very low prevalence of low birth weight (1.8%). Weight and length/height were measured within 10 days of the exact 1-month and 3-month anniversary of their birth, and within 15 days of the exact anniversary at 24 months of age. Body weight was measured to the nearest 50 g with electronic scales, with subjects wearing light indoor clothing. Recumbent length was measured to the nearest 0.1 cm using an infantometer at examinations before two years, whereas at the 24-month visit the height was obtained to the nearest 0.1 cm using a measuring tape attached to a wall. Both weight and length/height were taken in duplicate and means of the replicates were used in the analyses.

Socioeconomic and health-related variables were obtained from the PHB and from standardized observer-administered interviews. Type of birth delivery was collected from the PHB. Child sex, date of birth, parental age, parental occupation, and education level, as well as family structure and household income came from the interviews.

We examined the prevalence of overweight and obesity at two years of age, defined as BMI (kg/m^2^) equal to or greater than the 85^th^ and 95^th^ percentiles, respectively, of the age- and sex-specific curves from the World Health Organization (WHO) Child Growth Standards (WCGS) (2006) [24]. The WHO standards have been recommended for use in all children [24], and they have been used in many studies in different countries to characterize overweight and obesity in very young children for world-wide comparisons [5].

For the analysis, 3-month gains in weight and length were calculated by linear adjustment of the data at the measured intervals to exactly 3.0 months in duration (91 days). So, for example, for a child who weighed 3.0 kg at birth and 6.0 kg at the 3-month visit at 100 days of age, the adjusted 3-month weight = 6.0−(6.0–3.0 kg) ×(91/100)+3.0 = 5.73 kg. The length gains were similarly adjusted. Thereafter, adjusted BMI was calculated using the interval-adjusted weight and length. Preterm birth was defined as born prior to 37 weeks of gestation.

For analysis, maternal and paternal education levels were recorded into three categories (middle school, high school/technical, and university/college or higher). The occupations of parents were categorized into those typically reported in Chinese surveys. The monthly household income was recorded into ordinal categories from 1 to 6 (1 = <1,000 RMB, 2 = 1,000−<2,000 RMB, 3 = 2,000−<3,000 RMB, 4 = 3,000−<5,000 RMB, 5 = 5,000−<8,000 RMB, 6 = ≥8,000 RMB).

Although family structure was originally recorded as four nominal categories, i.e., 1 = nuclear family, 2 = extended family, 3 = joint family, 4 = single-parent family. Nuclear family refers to a family consisting of a pair of parents and their child(ren). Extended family refers to a nuclear family plus grandparents or siblings of the parents all living in the same household. Whereas, joint family is defined as the family consisting of 2 or more nuclear families with kinship. Single-parent family is the family where one parent lives with his/her dependent child(ren). Only categories of nuclear and extended families were used in the final regression models with categories 3 and 4 being excluded due to their very small numbers.

The reported occasions of bottle and breastfeeding were collected using a specially developed feeding questionnaire administered at monthly interviews with caregivers. This allowed categorization as “exclusive breast feeding,” “mixed feeding” and “formula feeding.” The feeding status at age two was classified in the first place into two groups, i.e., bottle-fed and non-bottle-fed. Thereafter, those bottle-fed children were further classified into three groups (never/rarely, sometimes, and mostly/always), according to the frequency of spontaneously milk/formula-emptying behavior and the behavior of empting milk/formula by encouragement of the caregiver.

For some variables there are small amounts of missing data so the actual number of cases may vary slightly in some descriptive summaries.

Chi-square tests were used to evaluate the weight status differences between sexes, analysis of variance was used to detect differences in mean BMI and BMI increments among different BMI groups, and the Student-Newman-Keuls (SNK) method was used for multiple comparisons of means. Differences between means for sex groups were compared by t-tests.

The hypothesis of this study is that early factors, such as parental weight status, infant birth weight, breastfeeding at the early stage of life, early weight increments and bottle-empty behavior at two years are related to the BMI status of children at two years of age. Logistic regression analyses were performed to test the hypothesis. Three logistic models are presented based on different grouping of BMI status used as the dependent variable. The use of extensive modeling is aimed to capture the above potential predictors effectively, which might not all be detectable in every single model due to the different grouping of BMI status. This approach is expected to be beneficial for the future from both the clinical perspective and public health alike, i.e., to develop more promising intervention and prevention strategies and programs.

For the first two models, binominal logistic regression models were developed with BMI status dichotomized as obese and other (obesity-only model) for model 1; and overweight and obese status combined in contrast with normal weight in model 2. A multinomial logistic approach was adopted in model 3, where BMI status at two years was modeled as a three-category variable (normal weight, overweight, obesity). Such factors as child gender, delivery type, gestational age, number of siblings, and parental social-demographic and economic variables were included as covariates in all three models. Level of statistical significance was set at p<0.05. The resulting odds ratios (OR) and 95% confidence intervals are presented. All data were analyzed using SPSS 10.0.

### Ethics Statement

This study has been approved by the Ethics Committee (EC) of Tongji Medical College, Huazhong University of Science and Technology (IORG0003571).

Written informed consents were obtained from the next of kin, caretakers, or guardians on behalf of the children participants involved in this study. The written informed consent form was approved by the EC.

## Results

Table 1 summarizes socio-demographic, perinatal and weight status of infants and their families. The BW for boys and girls are respectively 3.37±0.42 kg and 3.27±0.42 kg. Some of the variables highlight distinctive aspects of Chinese populations, e.g., high proportion of delivery by C-section (68%) and single-child families (87.4%), a very low prevalence of low birth weight (1.8%), and about 36% of children living in an extended family. Over 50% of parents have university or advanced degrees, indicating a well-educated parental background. The feeding patterns in the first month and the milk/formula-emptying behaviors at 2 years of age are also presented. There were 954 infants of 1098 (87%) reported to be bottle-fed.

**Table 1:** Variables describing the infants and their families in relation to children weight status at age two (N = 1098).

Variables		N(%)	NW	OW	OB	*P*
Boys (%)		563(51.3)	49.6	56.3	56.8	0.14
Number of siblings (%)						
0		960(87.5)	87.3	86.7	89.2	0.55
1		130(11.7)	11.7	13.3	10.8	
≥2		8(0.8)	1.0	0.0	0.0	
Birth weight (%)						
	Low birth weight (<2500 g)	20(1.7)	2.3	0.6	0.0	0.01
	Normal birth weight (2500∼<4000 g)	1006(91.8)	92.5	88.6	89.2	
	Macrosomia (≥4000 g)	72(6.5)	5.2	10.8	10.8	
Gestational age (%)						
	Preterm	43(3.9)	4.1	1.9	5.4	0.04
	Full term	1042(94.9)	95.2	96.2	91.0	
	Post term	13(1.2)	0.7	1.9	3.6	
Delivery mode (%)						
	Vaginal	352(31.7)	33.8	27.2	26.1	0.10
	C-section	746(68.3)	66.2	72.8	73.9	
Maternal age at child birth [mean (SD), yr]		1095	28.7±3.8	28.6±3.7	28.4±3.4	0.79
Paternal age at child birth [mean (SD), yr]		1091	31.4±4.8	30.9±4.4	30.8±4.2	0.23
Pre-pregnancy Maternal BMI [mean (SD), kg/m^2^]		1089	20.3±2.6	21.2±3.0	20.7±2.4	0.00
Paternal BMI [mean (SD), kg/m^2^]		1088	23.4±3.3	24.0±3.4	24.2±3.4	0.01
Maternal educational level (%)						
	Middle school or less (≤9 yr)	204(18.4)	18.7	17.1	19.8	0.23
	High school/technical (9–12 yr)	302(27.5)	2.9	31.6	18.9	
	University/college or advanced (>12 yr)	591(54.1)	53.4	51.3	61.3	
Paternal educational level (%)						
	Middle school or less (≤9 yr)	165(14.8)	16.1	10.8	13.5	0.43
	High school/technical (9–12 yr)	307(28.0)	28.2	27.2	27.0	
	University/college or higher (>12 yr)	625(57.2)	55.7	62.0	59.5	
Monthly household income (RMB, %)						
	<1000	29(2.7)	2.0	4.6	5.5	0.10
	1000-<2000	156(14.5)	13.3	16.9	19.3	
	2000-<3000	264(24.5)	24.0	26.6	23.9	
	3000-<5000	351(32.3)	33.1	27.3	33.9	
	5000-<8000	180(16.5)	17.7	14.9	11.0	
	≥8000	103(9.5)	9.9	9.7	6.4	
Family structure (%)						
	Single-parent family	5(0.5)	0.5	0.6	0.0	0.81
	Nuclear family	681(62.1)	62.2	63.9	57.7	
	Extended family	391(35.7)	35.2	33.6	41.4	
	Joint family	21(1.7)	2.1	1.9	0.9	
Feed pattern at 1st month (%)						
	Exclusive breastfeeding	367(37.1)	38.3	34.8	29.6	0.47
	Mixed feeding	486(48.9)	48.3	49.3	54.1	
	Formula feeding	139(14.0)	13.4	15.9	16.3	
Bottle feeding at two years (%)		954(86.9)	87.0	84.2	90.1	0.36
Children spontaneously milk/formula-empty behavior at age two (%)						
	Never/rarely	71(7.5)	8.1	9.0	1.0	0.06
	Sometimes	109(11.4)	11.2	14.3	9.0	
	Mostly/always	774(81.1)	80.7	76.7	90.0	
Children emptied milk/formula by encouragement at age two (%)						
	Never or rarely	241(25.4)	27.0	24.1	14.0	0.08
	Sometimes	567(59.4)	58.4	59.4	67.0	
?	Mostly/always	146(15.2)	14.6	16.5	19.0	

Notes: NW- normal weight (BMI<P_85_), OW-overweight (P_85_≤BMI<P_95_), OB- obese (BMI≥P_95_),.

Table 2 presents mean child BMI and frequency of BMI status at two years of age, as well as the mean BMI increments in the first three months. Compared with girls, boys have greater mean BMI in both normal and overweight groups. However, this gender difference was not observed in the obese group. The overweight and obese boys tend to have greater BMI increments in the first three months than their normal-weight counterparts. Interestingly, for girls the overweight group appeared to have a slightly lower mean BMI increment than normal-weight peers, while obese girls’ BMI increment ranked the highest. The prevalences of combined overweight and obesity in boys and girls at two years was 27.0% and 21.9%, respectively, without a significant statistical difference between the sexes.

**Table 2:** Children’s BMI at age of two, BMI increments in the first three months in relation to their weight status at two years, and overweight and obesity prevalences at two years of age.

Weight Status at 2 years	BMI at 2 years of age [mean (SD), kg/m^2^]		ΔBMI [mean (SD), kg/m^2^]		n (%) #	
	Boys	Girls	Boys	Girls	Boys	Girls
Normal Weight	16.08 (0.85)	*15.74 (0.79)	4.25(1.75)	3.92(1.75)	411 (73.0)	418 (78.1)
Overweight	17.76 (0.30)	*17.48 (0.32)	4.87(1.62)	3.46(1.90)	89 (15.8)	69 (12.9)
Obese	19.07 (0.79)	19.01 (1.05)	4.63(1.45)	4.19(2.35)	63 (11.2)	48 (9.0)
Total	16.68 (1.31)	*16.26 (1.29)	4.38(1.72)	3.89(1.82)	563 (100.0)	535 (100.0)

Note:.

Δ BMI = BMI at 3^rd^ month – BMI at birth.

* t test with significant gender difference.

# Chi-square test with *χ^2^ = *3.91* p* = 0.14.

Table 3 presents the crude and adjusted odds ratios from the three logistic regression models, using different combinations of weight status. Born with macrosomia, greater BMI increment in the first three months, and children’s bottle emptying by encouragement were identified as significant risk factors for higher weight status across the three models. The children’s spontaneous bottle emptying at the age of two was associated with the obesity status (model 1), but not in the other two models. Pre-pregnancy maternal and paternal BMI appeared to have significant impacts on their offsprings’ weight status with one unit increase (1kg/m^2^) of BMI slightly increasing the risk of children becoming overweight or obese (BMI≥P_85_) by 12% and 6%, respectively in model 2.

**Table 3:** Crude and adjusted odds ratios (OR) and 95% confidence intervals (CI) for overweight and obesity at 2 years old.

Models		Model 1		Model 2		Model 3	
Classifications of outcome variable - weight status at two years of age		1 -obese (BMI≥P_95_)		1- overweight+obese (BMI≥P_85_)		2 -obese (BMI≥P_95_)	
		0- non-obese (BMI<P_95_)		0- normal weight (BMI<P_85_)		1- overweight (P_85_≤BMI<P_95_)	
						0- normal weight (BMI<P_85_)	
**Predictors**		**Crude Odds Ratio** **(95% CI)**	**Adjusted Odds Ratio** **(95% CI)**	**Crude Odds Ratio** **(95% CI)**	**Adjusted Odds Ratio** **(95% CI)**	**Crude Odds Ratio** **(95% CI)**	**Adjusted Odds Ratio** **(95% CI)**
Birth weight (ref: BW <4000 g)		1.37 (0.99, 1.90)	1.80 (1.20, 2.70)	1.49 (1.16, 1.90)	1.88 (1.38, 2.58)	1.46 (1.15, 1.86)	1.85 (1.37, 2.51)
Pre-pregnancy Maternal BMI		1.03 (0.96, 1.11)	1.00 (0.90, 1.12)	1.10 (1.04, 1.15)	1.12 (1.05, 1.20)	1.09 (1.03, 1.14)	1.09 (1.02, 1.17)
Paternal BMI		1.06 (1.01, 1.13)	1.03 (0.96, 1.12)	1.06 (1.02, 1.11)	1.06 (1.01, 1.12)	1.06 (1.02, 1.11)	1.06 (1.01, 1.12)
Feeding pattern at 1st month							
(ref: formula feeding)							
	Exclusive breastfeeding	0.66 (0.35, 1.26)	0.56 (0.25, 1.25)	0.71 (0.45, 1.11)	0.53 (0.31, 0.90)	0.70 (0.45, 1.10)	0.53 (0.31, 0.90)
	Mixed breastfeeding	0.94 (0.52, 1.70)	0.78 (0.37, 1.63)	0.88 (0.58, 1.35)	0.82 (0.50, 1.35)	0.89 (0.58, 1.35)	0.81 (0.50, 1.33)
BMI increment in the first three months [kg/m^2^]		1.11 (0.97, 1.27)	1.16 (1.02, 1.32)	1.09 (0.99, 1.19)	1.15 (1.04, 1.27)	1.09 (0.99, 1.20)	1.16 (1.05, 1.29)
Children-initiated bottle emptying* at age two (ref: rarely)		1.88 (1.23, 2.88)	2.31 (1.24, 4.31)	1.10 (0.87, 1.39)	1.20 (0.82, 1.77)	1.15 (0.91, 1.46)	1.25 (0.85, 1.85)
Bottle emptying by encouragement (ref: rarely)		1.53 (1.09, 2.15)	1.57 (1.06, 2.23)	1.31 (0.97, 1.77)	1.30 (1.03, 1.64)	1.32 (1.05, 1.67)	1.35 (1.02, 1.80)

Notes: Adjusted for child gender, delivery type, gestational age, number of siblings, maternal age, parental occupation and education level, household income, family structure and if fed by bottle.

* Bottle-emptying behavior describes that children finished all the milk or formula made available to them (spontaneously or by encouragement) offered in a bottle, regardless of the volume.

Multinomial logistic analysis in model 3 showed that a one unit increment in pre-pregnancy maternal BMI raised, on average, 9% the risk of children becoming overweight, or from overweight to obese at two years of age. Similarly, impact was observed in paternal BMI with a reduced risk of 6%. Interestingly, the results also demonstrated the long-term effect of exclusive breastfeeding (EBF) at the first month on the weight status at two years of age. In contrast with formula-fed infants, those fed exclusively breast milk were at a lower risk of high BMI status, i.e., model 2 showed 47% (CI = 10%–69%) less likely to develop overweight and obesity by the age of two. The significant protective effect of EBF at the first month was also evidenced in model 3. However, a similar effect was not shown in the mixed-feeding group.

## Discussion

Childhood BMI is known as a strong predictor of adult obesity [25], [26]. The age of two years of age appears to be a crucial time because many obesity-related life-style habits are formed at this early stage, and thereafter are very likely to track into later life [27]. This current prospective study should contribute to a deeper understanding of the potential contributors, especially some modifiable risk factors, to help develop more effective prevention strategies targeting the emerging childhood obesity problem in China.

In this study, almost one-fourth of children at age two had BMI equal to or exceeding the 85th percentile of the WHO Growth Standard. The prevalences of overweight and obesity were 14.38% and 10.11%, respectively. These numbers are similar to the reported prevalences of overweight and obesity (10.9% and 13.8%) in years 2008–2009 in six Northeast China cities relative to the WHO Growth Standard [28], but in a 3-year older population than the present study. The present results are also comparable to the obesity statistics in some Western countries [5], [29], [30].

We investigated the association between breastfeeding and weight status. The any-breastfeeding rate in the first month was 86%, with 37% of infants being exclusively breastfed (EBF), and the remaining 49% fed on a combination of breast milk and formula. The observed EBF rate in our sample is relatively lower than the full breastfeeding rate in Beijing (56.6%, in 2002) [31]. In contrast with the statistics from the US in 2002, i.e., 42.5% of infants being exclusively breastfed, and 51.5% partially breastfed [32] at 3 months, the corresponding rates from our study were a little lower even though our study subjects were younger. The role of breastfeeding in controlling the epidemic of obesity has long been controversial [33], [34], although its effect on the promotion of the health of both mothers and children is universally recognized. In the current cohort, interestingly, a concurrent protective effect and a two-year impact of breastfeeding were both observed. The former was reported in our previous paper [23] regarding high weight gain and high BMI status in the first three months, showing that a higher breastfeeding proportion at 3 months was significantly associated with the reduction of the risks of high weight gain and high BMI status, although this effect was not observed from breastfeeding proportion at the first month of age. The protective effect of breastfeeding during the first month on the children’s weight status at age two was observed in adjusted regression models, with weight status dichotomized or treated as a three-category variable. The results showed that exclusive breastfeeding reduced the risk of overweight and obesity by 47% at age two, however mixed-feeding was not shown to be protective. Haisma et al [35] suggested that protective effects of EBF on subsequent obesity might result from a more appropriate energy intake associated with it.

Our results again emphasize that early initiation of breastfeeding and encouragement of exclusive breastfeeding have potentials to play important roles regarding obesity prevention from the early years of life. Breastfeeding appears to be a personal decision and behavior; however, in addition to parental knowledge level, behaviors and preference, there are many other factors that inhibit the promotion of EBF, including marketing activities of infant formula through hospitals [36], and Caesarian section (C-section) [37], [38], etc. Therefore, a supportive household as well as social environment, e.g., education targeting new parents and their families, and policy-based and multi-disciplinary intervention strategies including advocacy may be vital for this decision to become a successful reality and for breast feeding behavior to be sustained.

The impact of parental BMI and birth weight was also investigated in the present study. We observed that one unit paternal and pre-pregnancy maternal BMI change raised, on average, 6% and 9% the risks of children becoming overweight or from overweight to obese at two years of age. This is consistent with previous studies, i.e., Katrinna, et al reported that having 2 overweight parents was associated with an increased risk of child obesity [odds ratio (OR): 2.2]. [39] This association might be somewhat due to shared genetic backgrounds between parents and their offpring, but it could also be related to parents’ different concepts regarding child growth and feeding, as well as the actual feeding behaviors given their own BMI status.

In the present study, the children born with macrosomia were 80% to 85% more likely to become overweight and/or obese at two years of age. This was also observed as a strong predictor for BMI category at one year of age (odds ratio, 4.38) elsewhere [40]. The Chinese people in general expect a big baby to be born. In our sample, the percentage of low birth weight was very low (1.8%), but that of macrosomia was comparative higher (6.4%), especially in the overweight and obese group of children (10.8%). Population increases in birth weight do not necessarily lead to improved health, as the U-shaped impact of birth weight on health has been documented, with small size at birth associated with subsequent cardiovascular disease and diabetes, and large size associated with obesity and cancer [41]. Birth weight is generally considered as a gauge of nutritional status of the mothers pre-pregnancy and then of gestational nutritional status. However, in our study the BW effect appears to be dependent on parental BMI, with almost identical odds ratios in the regression model without parental BMI (1.93) and in the full model (1.85).

Previous studies have demonstrated that rapid infancy weight gain (RWG) is an important indicator for subsequent obesity at different ages, using various definitions of RWG [42], [43]. We examined the impact of the BMI increment during the first three months on the weight status at age two in this study in different ways, i.e., with or without birth weight included in the regression models. The results showed that this early BMI increment is a robust predictor for weight status at age two, although the odds ratios vary slightly. Hence, we firmly believe from the perspective of early childhood obesity prevention, the BMI increment needs to be a focus of attention of caretakers and child health care practitioners. Some of the variation in early gain in BMI is probably related to household factors. It was shown in our previous paper that lower breastfeeding proportion increased the risk of high BMI gain in the first three months [23].

In the present study the adjusted models demonstrated that the children who by caregiver encouragement, often or sometimes emptied milk/formula were at a greater risk (30%–53%) of overweight and/or obesity at age two, compared with their counterparts who never or rarely did so. In Chinese culture, people traditionally believe that chubby babies are healthy babies, and they consider serving huge amounts of food or encouraging children to eat as symbols of showing their love. The society admires and appreciates those caretakers who raise a heavier baby and the population believes that they are demonstrating high levels of parenting quality and competence.

The association of food with love in some cultures may also contribute to higher infant weight [44]. This perception tends to result in such feeding behaviors as encouragement, pushing and prompting. For instance, according to Jiang’s qualitative research, grandparents tended to urge children to eat more meals and larger portions at served meals for preschool children [45]. Clark’s study reported that children-feeding behaviors among parents directly influence children’s food intake and weight [46]. By encouragement, the children might consume more food than needed, thus leading to extra energy intake and thereafter gaining extra weight. These attitudes and behaviors of caretakers might have greater effects among younger children because they are more attached to and rely on their caretakers, and may be more obedient than older children. For children from birth to 2 years in China, almost all the food children consume, either homemade or commercially available, is provided at home.

Our results regarding the role of encouragement conflict with those of Li R’s study, who found the mothers' encouragement of bottle emptying was negatively associated with their infants' risk for excess weight during the second half of infancy [47]. The inconsistency may be related to the age difference in the study populations, the level of parents’ understanding of what was considered as the behavior of “encouragement” and actual encouragement behaviors. Nevertheless, as a crucial component in the development of childhood nutritional habits with undeniable influence on their weight status, the family feeding behaviors are a concern in early childhood obesity intervention strategies.

Interestingly, the children-initiated bottle emptying was also observed with striking impact on weight status in our study, but only in the model comparing the obese children with all others (model 1). We suspect these inconsistent results across the models can be at least partially due to the different classification of weight status as well as the distribution of children-initiated bottle emptying among different BMI groups. We believe that the child-initiated bottle emptying behavior reflects children’s appetite and eating speed. Certainly more detailed studies are needed for a better understanding of the relationships between child-initiated bottle emptying behaviors and early childhood obesity.

Some commonly reported factors with effects on overweight were not observed in our population, such as child’s gender, maternal education level, and household economic status. A gender difference can be observed in the raw statistics of overweight and obesity prevalence, with boys at higher risk than girls; however, this difference did not appear in the multivariable regression models. We believe that in single-child families, the attitudes and the ways of parenting of the parents tend to be rather homogeneous, i.e., with high expectations for their only child irrespective of gender. In our study the parents tend to be well educated, with about 85% of parents receiving 12 years of education, and over 50% of them having university or advanced degrees. The education level of parents in our study population might not be diverse enough to enable detection of significant education impacts; similarly, with household income, because it is highly related to education level.

### Limitations

We used the WHO Children Growth Standard (WCGS) as the reference of weight status, which does not contain any East Asian populations which might affect the results, to the degree that genetic or population-specific factors play roles in early growth patterns. Nevertheless, because we are investigating interrelationships among variables associated with overweight/obesity status, it is unlikely that the relationships are misrepresented.

The birth weight and crown-heel length were obtained from the Perinatal Health Booklet (PHB) which were obtained from regular child heath visits and collected at local clinics. These data might not be as precise as the data obtained under more carefully controlled research settings. There is no reason to suspect, however, that such data are biased even though they may be less reliable. Consequently any associations found related to the birth variables, e.g., early weight gain are probably underestimates of the true associations.

The reduction of available measurements as the older child visits was comparatively high. We anticipated this situation so that we over-recruited infants initially as planned to allow enough statistical power at the final visits. Furthermore, we tested the social demographic and birth variables between the initially recruited and remaining participants without significant differences detected except for a small difference in the mean age of fathers (31.3 vs. 31.0 years). Consequently, we believe study attrition should not have appreciable impact on the generalizability of our results.
